# Copepod reproductive effort and oxidative status as responses to warming in the marine environment

**DOI:** 10.1002/ece3.8594

**Published:** 2022-02-18

**Authors:** Ella von Weissenberg, Anna Jansson, Kristiina A. Vuori, Jonna Engström‐Öst

**Affiliations:** ^1^ 8058 Tvärminne Zoological Station University of Helsinki Hangö Finland; ^2^ Novia University of Applied Sciences Ekenäs Finland; ^3^ 8058 Laboratory of Animal Physiology Department of Biology University of Turku Turku Finland; ^4^ 8058 Department of Equine and Small Animal Medicine University of Helsinki Finland

**Keywords:** climate change, marine ecology, oxidative stress, trade‐off, zooplankton

## Abstract

The marine ecosystems are under severe climate change‐induced stress globally. The Baltic Sea is especially vulnerable to ongoing changes, such as warming. The aim of this study was to measure eco‐physiological responses of a key copepod species to elevated temperature in an experiment, and by collecting field samples in the western Gulf of Finland. The potential trade‐off between reproductive output and oxidative balance in copepods during thermal stress was studied by incubating female *Acartia* sp. for reproduction rate and oxidative stress measurements in ambient and elevated temperatures. Our field observations show that the glutathione cycle had a clear response in increasing stress and possibly had an important role in preventing oxidative damage: Lipid peroxidation and ratio of reduced and oxidized glutathione were negatively correlated throughout the study. Moreover, glutathione‐s‐transferase activated in late July when the sea water temperature was exceptionally high and *Acartia* sp. experienced high oxidative stress. The combined effect of a heatwave, increased cyanobacteria, and decreased dinoflagellate abundance may have caused larger variability in reproductive output in the field. An increase of 7°C had a negative effect on egg production rate in the experiment. However, the effect on reproduction was relatively small, implying that *Acartia* sp. can tolerate warming at least within the temperature range of 9–16°C. However, our data from the experiment suggest a link between reproductive success and oxidative stress during warming, shown as a significant combined effect of temperature and catalase on egg production rate.

## Graphical abstract

The aim of this work was to study warming effects on zooplankton reproduction and oxidative status and seek potential trade‐offs. We found that glutathione cycle was effective in preventing oxidative damage during warming, although warming had a negative impact on reproduction. Our data suggest a possible trade‐off between reproduction and oxidative status during warming.

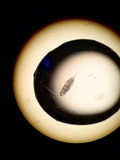

## 1 INTRODUCTION

Oxidative stress is a condition, which occurs when the production of reactive oxygen species (ROS) in cells exceed the antioxidant defense and repair capacity, causing serious damage to DNA, lipids, and proteins (Costantini, 2008; Hulbert et al., 2007). ROS production is tightly linked with aging and lifespan, as oxidative damage tends to accumulate in aging animals (Finkel & Holbrook, 2000). Furthermore, antioxidant defense and repair mechanisms have been considered to have a trade‐off with reproduction effort: Reproduction may increase oxidative stress, and the stress levels tend to increase with higher effort in offspring quantity and quality (Metcalfe & Alonso‐Alvarez, 2010). Aging has also been shown to decrease egg quality and production in invertebrates (Giron & Casas, 2003; Powers et al., 2020; Rodríguez‐Graña et al., 2010). ROS production can increase due to external stress, such as temperature and salinity change, intense UV light, acidification, and toxins (Lesser, 2006; Lushchak, 2011). Elevated temperature in particular is a relevant environmental factor affecting oxidative status, as it stimulates metabolism and may enhance ROS production via increased oxygen consumption. Especially, ectotherms are influenced by temperature changes in the environment (Lushchak, 2011).

Copepods are an important link between primary producers and higher trophic levels. They experience strong environmental variability on daily, seasonal, and annual scale, as their diel vertical migration behavior exposes them to a large gradient of physico‐chemical conditions, such as temperature, salinity, and pH (Almén et al., 2014; Engström‐Öst et al., 2019; Lewis et al., 2013). Thus, copepods have traditionally been considered fairly robust to environmental changes, but can be quite sensitive to thermal changes (Garzke et al., 2020; Vehmaa et al., 2013), whereas are less sensitive to pH and slight ocean acidification (Engström‐Öst et al., 2019, 2020; Niehoff et al., 2013). UV light can also cause DNA damage and oxidative stress in zooplankton, especially in clear waterbodies (Tartarotti et al., 2014). In the Baltic Sea, harmful UV light is absorbed in the surface layer of the sea. Depending on the chlorophyll concentration, visible light, including UV, is almost completely absorbed between 0.7 and 3 m depth (Dera & Wozniak, 2010). UV light can therefore be an important factor causing eco‐physiological changes in copepods, but potential damage is highest in the sea surface of the Baltic Sea.

Elevated temperature tends to favor smaller plankton over large ones, a phenomenon that has ecological consequences in the whole marine ecosystem: The whole community is shifting to smaller size starting from primary producers, and the mean body sizes are decreasing on species level (Daufresne et al., 2009). Elevated temperature causes northward shift of copepod species in the Northern Hemisphere (Beaugrand et al., 2002). Furthermore, associated increase in warmwater species and decrease in coldwater species were recorded already 20 years ago in the North Atlantic (Beaugrand & Reid, 2003). Mäkinen et al. (2017) analyzed long‐term data from 1967 to 2013 in a coastal area in southwestern Finland and demonstrated a temperature‐ and salinity‐related decline of large calanoid copepods and increase of smaller‐sized brackish taxa. Adult copepods have also reduced in size and abundance due to warming (Garzke et al., 2016). Copepods are expected to grow faster but mature at smaller size in higher temperature due to temperature‐size rule (Atkinson, 1994; Bergmann, 1847), which may have negative consequences on egg production. On the other hand, increase in temperature is shown to increase egg production rate and egg hatching success in *Acartia* copepods (Peck & Holste, 2006). Also an experimental study of Vehmaa et al. (2012) indicated that *Acartia* sp. females were able to match the phenotype of their eggs to the new environment.

The sea surface temperature (SST) in the Baltic Sea is predicted to increase by almost 2°C during the 21st century (Graham, 2004; HELCOM, 2021; Meier et al., 2012), and within 85 years, the SST has already increased by 1°C in the western Gulf of Finland (Merkouriadi & Leppäranta, 2014). In order to reveal temperature effects on oxidative stress, either antioxidant or oxidative stress biomarkers can be used as physiological measures of oxidative status. Glutathione (GSH) is an antioxidant that has an important role in preventing cell damage by ROS; GSH reduces peroxides during acute oxidative stress and oxidizes; and the glutathione cycle reduces GSSG back into GSH. Thus, the ratio of reduced (GSH) and oxidized (GSSG) glutathione can be calculated and used as an indicator of oxidative stress (Lesser, 2006). Glutathione s‐transferase (GST) contributes to catalyze reactions between GSH and peroxides, and may even help in protection against lipid peroxidation. Catalase (CAT) is an antioxidant that scavenges hydrogen peroxide (H_2_O_2_) and catalyzes its conversion to O_2_ and water (Halliwell & Gutteridge, 2015). Oxygen radical absorbance capacity (ORAC) is used as a measure of antioxidant capacity (Prior et al., 2003). Oxidative stress can also be interpreted from oxidative damage on biomolecules, such as lipids, proteins, and DNA. Lipids are susceptible to damage caused by peroxides (such as H_2_O_2_), a condition called lipid peroxidation (LPX) (Halliwell & Gutteridge, 2015).

The biomarkers described above (LPX, CAT, GSH:GSSG, GST, and ORAC) are commonly used for studying oxidative stress in zooplankton (Cailleaud et al., 2007; Souza et al., 2010; Vehmaa et al., 2013). Copepods have an effective glutathione metabolism, which makes them more capable of dealing with excess ROS production (Glippa et al., 2018; Vuori et al., 2015). Nevertheless, previous studies have shown that oxidative stress has a negative effect on viable egg production in calanoid copepods (Garzke et al., 2016), whereas warming has a negative effect on oxidative status in copepods (Glippa et al., 2018; Kim et al., 2015; Won et al., 2015).

The aim of this work was to study the relationship between offspring production rate and oxidative stress response of a key copepod species *Acartia* sp., both in the field over the whole productive season, and in an experimental setup using different temperatures. We hypothesized that the production of ROS exceeds the antioxidant defense in higher temperatures, causing oxidative stress. Furthermore, we expected to find a trade‐off between oxidative status and offspring production at elevated temperatures, suggesting increasing costs of reproduction due to warming (Vehmaa et al., 2013).

## 2 MATERIALS AND METHODS

### 2.1 Field sampling

**FIGURE 1:**
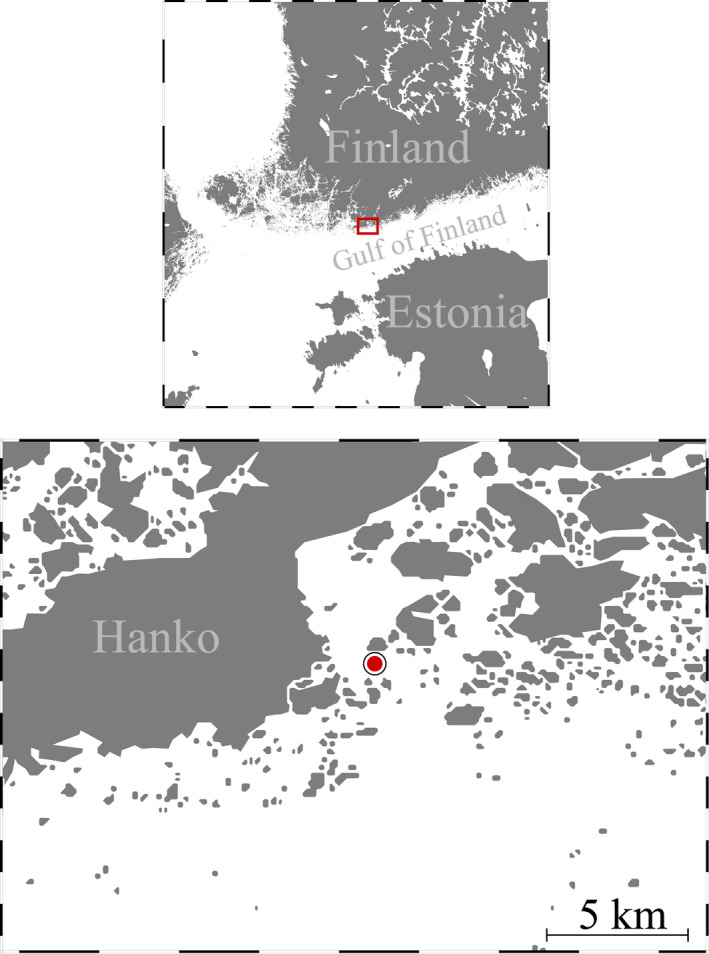
Map of the study area in Hanko, Finland. The red dot shows the sampling site in Storfjärden, which is close to Tvärminne Zoological Station. Ocean Data View was used for creating the map (Schlitzer, 2016)

Water samples and zooplankton were collected bimonthly, in total six times between May and August 2018. Additional sampling was conducted three times in June for the experiment. Sampling took place in a pelagic area Storfjärden (59°52′56″N, 23°15′14″E), close to the Tvärminne Zoological Station in the southwestern Gulf of Finland (Figure 1). CTD (conductivity, temperature and depth) data were obtained from Tvärminne Zoological Station monitoring series. Oxygen and temperature were measured at every five m until 30 m depth during each sampling occasion, using YSI pro ODO oxygen sensor. One water sample was collected at 5, 10, and 15 m depth using a 5 L Limnos water sampler. From each depth, samples for pH were carefully collected in 250‐ml glass bottles without airspace. Chlorophyll *a* (Chl *a*) water samples were collected from the same depths. One 40 ml phytoplankton sample was obtained by mixing 5 L Limnos samples collected at 5, 10, and 15 m depth and by filtering the water through a 10 m plankton net.

#### 2.1.1 Zooplankton

In order to collect copepods for the *in situ* egg production and oxidative stress analyses, three zooplankton samples were taken between 30 m depth and the surface using a 200 m plankton net with cod‐end, and emptied into a cooler with seawater from 10 m (Engström‐Öst et al., 2015). Zooplankton was kept in a climate chamber at ambient sea water temperature until sorting commenced. The animals were always used during day of sampling, usually within a few hours. During each sampling occasion, 50 adult female *Acartia* sp. copepods were sorted using glass Pasteur pipettes and incubated in 250‐ml false bottom chambers (*N* = 5, 10 females/chamber, mesh size: 120 m) containing 1.2 m filtered seawater (FSW) at ambient temperature (approximately 10 m depth) in a climate controlled room. The main *Acartia* species occurring in the area is *Acartia bifilosa*, but *A*. *tonsa* is present especially in late summer (Almén et al., 2014; Engström‐Öst et al., 2015; Katajisto & Viitasalo, 1998; Katajisto & Viitasalo, 1998). *Acartia* females were unfed to obtain egg numbers produced from past resources. In Finiguerra et al. (2013), total egg production and survivorship during starvation were uncorrelated in *Acartia tonsa*. After 24 h, females, eggs, and hatched nauplii were separated by sieving and counted, and the females were conserved with acid Lugol's solution for body size analysis. Acid Lugol's solution can affect body size to some extent (up to 17% in copepods) (Jaspers & Carstensen, 2009), which needs to be considered when comparing the body sizes in this study to those reported in studies using other conservation methods.

The number of eggs, nauplii, and live females was used for calculating *in situ* egg production rate (eggs female^−1^ d^−1^). Additionally, at each sampling occasion, 30 females (*N* = 5) were picked by forceps into 1.5‐ml Eppendorf tubes, snap‐frozen in liquid *N,* and stored at −80°C for biomarker analysis.

#### 2.1.2 Measurements

In the laboratory, pH was measured using a WTW inoLab series pH meter. Chl *a* samples were processed by filtering 100 ml of seawater using 25‐mm glass fiber filters (Whatman GF/C). The filters were submerged in 10 ml of ethanol (96%) and stored at −20°C, and determined by fluorometry (Varian Cary Eclipse Fluorescence Spectrophotometer), using a 96‐well microplate reader. Each 40 ml phytoplankton sample was treated with acid Lugol's solution and stored at 3°C. The samples were analyzed semiquantitatively by counting the individuals and using an Utermöhl sedimentation chamber. Corresponding phytoplankton groups were identified and their size measured using a 40× and 20× magnification with a microscope (Leica). Ten phytoplankton groups were monitored, of which diatoms, chlorophytes, chrysophytes, cyanobacteria, dinoflagellates, and prasinophytes were most common. As the phytoplankton sample was run through a 10 m net, microalgae <10 m are missing.

### 2.2 Experimental setup

**FIGURE 2:**
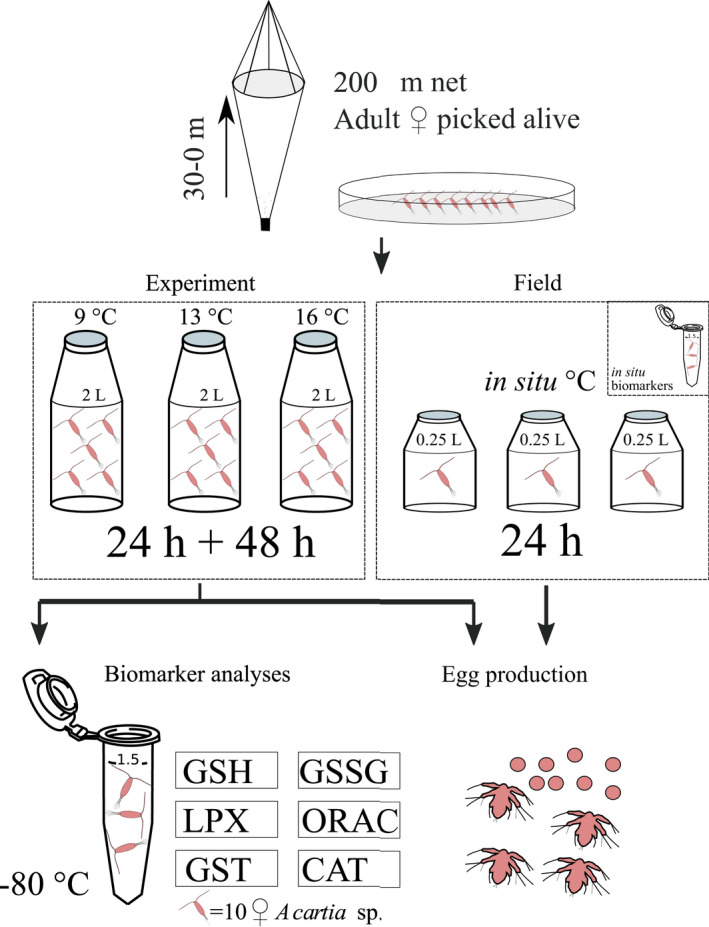
Study design of field monitoring and experimental setup. Adult *Acartia* sp. were collected with a 200‐m net, then sorted and picked under a microscope for incubation (experiment: 24‐h acclimation and 48 h in the experiment; field monitoring: 24 h). The eggs and nauplii were counted from the sample, and 30 females were picked for GSH, GSSG, GST, CAT, ORAC, and LPX analyses

In order to evaluate copepod reproductive output and oxidative stress, female copepod *Acartia* sp. were incubated for egg production, hatching, survival, and oxidative stress measurements in three temperatures: control temperature 9°C, and two elevated temperatures 13°C and 16°C (Figure 2). The 9°C treatment represents the seawater temperature at 10 m depth prior to the experiment. The two other temperatures represent a projected increase by 2100 according to IPCC RCP 8.5 (current emission trajectory) model prediction (IPCC, 2014). Copepods for the experiment were collected in similar manner as described in Section 2.1, and sampling was conducted on three occasions: 14, 16, and 21 June 2018. Female *Acartia* sp. were gently sorted with glass pipette and transferred to 2.2‐L bottles (*N* = 3) containing 1.2 m FSW for each temperature treatment (ca. 50 ind. Bottle^−1^). A few males (5–6 ind.) were added to each bottle (Engström‐Öst et al., 2015; Vehmaa et al., 2012, 2013). Nevertheless, there may be potential male bias in the data, as we were not able to check the male reproductive stage during sorting. FSW was produced by filtering seawater through a 10‐m plankton net, and subsequently through GF/C glass fiber filters (47 mm, Whatman). The conditions in the field during the experiment were monitored as mentioned above (see Field sampling). The bottles were incubated in three different climate chambers, set at 9°C, 13°C, and 16°C. To keep the water temperature as stable as possible, the bottles were incubated in water baths. The copepods were fed once daily with a commercial high‐quality solution consisting of *Isochrysis*, *Pavlova*, *Tetraselmis*, *Thalassiosira pseudonana*, and *T*. *weissflogii* (Shellfish diet 1800, Reed Mariculture) with a final concentration of 10,000 ml^−1^, which corresponds to a subsaturated food concentration for copepods (Klein Breteler and Gonzalez, 1982).

The experiment lasted 72 h in total: 24‐h acclimation (Dutz & Christensen, 2018; Vehmaa et al., 2013) and 48‐h experiment. The bottles were slowly stirred a few times per day. Dissolved oxygen (mg L^−1^) was measured daily, and copepod condition monitored. After the experiment, the bottle was emptied through a 200‐m mesh, and copepod survival, number, and condition were checked under a stereo microscope (Leica). The water was rerun through a 48‐m mesh to collect eggs that were transferred to a petri dish. The eggs were counted and set to hatch in the same temperature as they were produced. Thirty females in good condition were transferred to Eppendorf tubes for biomarker analysis (similarly as mentioned above). All remaining live females in each replicate were preserved in an Eppendorf tube with acid Lugol's solution for body size measurements. Prosome length P*_L_* was measured under a microscope Leica MZ12 attached to Nikon DS‐L3 camera.

### 2.3 Biomarker analyses

The *Acartia* samples (field and experiment) were analyzed for oxidative status biomarkers (Glippa et al., 2018; Vuori et al., 2015) in the Laboratory of Animal Physiology, University of Turku, Finland. The analyses were carried out according to protocols in Vuori and Kanerva (2018a, 2018b, 2018c, 2018d, 2018e). Concerning Oxygen Radical Antioxidant Capacity ORAC Activity Assay (Cell Biolabs), we used the assay kit protocol. Zooplankton samples were entirely homogenized in 100 L of 0.1 M K2HPO4 + 0.15 M KCl buffer (pH 7.4) using a Tissue Lyser II bead mill (Qiagen). An aliquot of raw homogenate (25 L) was immediately frozen in liquid *N* and stored at −80°C for lipid peroxide determination (LPX). Then, the sample homogenate was centrifuged at 10,000*g* for 15 min at 4°C and the supernatant was divided into aliquots for glutathione‐s‐transferase (GST), catalase (CAT), and ORAC assay and for glutathione sample preparation. The glutathione sample was deproteinized by adding 5% sulfosalicylic acid (SSA) and subsequently incubated on ice for 10 min., and centrifuged for 10 min. at 10,000 *g* at 4°C. The supernatant was divided into two different tubes for reduced (GSH) and oxidized glutathione (GSSG), and 33 mM M2VP (1‐methyl‐2‐vinylpyridinium trifluoromethanesulfonate, Sigma Chemicals) in 0.1 M HCl, a scavenger of GSH, was added to the GSSG sample. The sample homogenate aliquots and glutathione samples were frozen in liquid *N* and stored at −80°C until analysis.

### 2.4 Statistical analyses

Statistical analyses were conducted using free software R, version 3.6.1, R Core Team (2019). Differences in the mean P*_L_* between treatments of the experiment and the sampling days were tested using a Kruskal–Wallis rank sum test. A Spearman correlation test was used when testing any correlations. Linear mixed models were carried out by using the lmer function in the *lmerTest* package in R (Kuznetsova et al., 2017). The assumption of LMM, that is, the normality of model residuals, was assessed by use of the Shapiro–Wilk test. Log transformation was made for ORAC and *in situ* GST activity to gain better fit for the data and linear response. In all models, temperature treatment (experiment) or *in situ* temperature from 10 m depth (field) was used as a fixed effect and sampling date as a random effect. Egg production rate was used as a response variable, and each biomarker separately as a second fixed effect, sometimes in interaction with temperature. Additionally, temperature effects on biomarkers were tested; in these models, biomarkers were used (separately) as response variables. The Akaike Information Criterion (AIC) was used for model selection; models having the lowest AIC were selected. Interaction (x) between treatments and biomarkers was used only when the model had a smaller AIC value than the model without interaction. All biomarkers were also separately used as a response variable. One incubation bottle was excluded from the data as an outlier due to low egg production rates.

## 3 RESULTS

### 3.1 Environmental conditions

**FIGURE 3:**
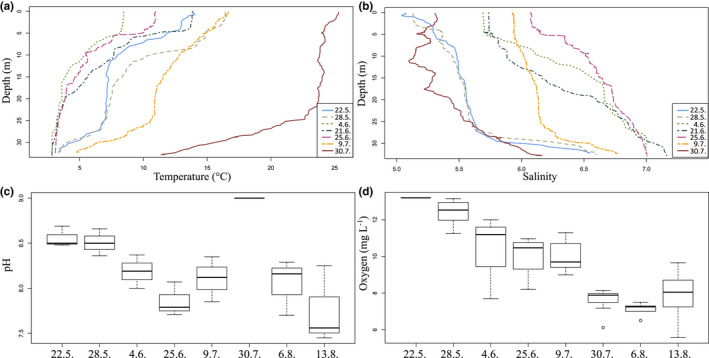
CTD profiles of (a) temperature and (b) salinity in all sampling dates, (c) pH values and (d) oxygen concentration (mg L^−1^) during the season. Oxygen and pH data were derived from 10 m depth. The boxplots (c, d) show the median (vertical line), interquartile range (IQR, the box), and minimum and maximum within 1.5 × IQR (“whiskers”) and outliers (circle)

The thermocline at Storfjärden occurred below 5 m in June but descended to approximately 25 m in mid‐July. The surface water temperatures were between 8 and 11°C in May–June, while the bottom temperatures remained below 5°C (Figure 3a). The temperature increased steeply between July 9 and 30; it remained between 22 and 25°C down to the thermocline, and the bottom temperature was as high as 12°C. The salinity within the water column varied between 5.1 and 7.2 throughout the season (Figure 3b). Temperature correlated negatively with salinity (−0.71, *p* < .01) and oxygen concentration (−0.68, *p* < .01), and positively with pH (0.74, *p* < .01) at 10 m depth. The temperature and salinity data are missing for June 14 and 16.

In general, pH at 10 m depth was higher in May than in August (Figure 3c). However, the peak in pH (8.7) was reached on July 30, and the lowest measurement (7.5) was recorded August 13. The oxygen concentration at 10 m depth was highest in May (13.2 mg L^−1^) and decreased toward the end of the sampling season, the minimum being 6.5 mg L^−1^ on August 6 (Figure 3d). Average dissolved oxygen was 10.1 ± 2.01 g L^−1^ over the season.

### 3.2 Food conditions—Phytoplankton community structure (>10 m) and Chl *a*

**FIGURE 4:**
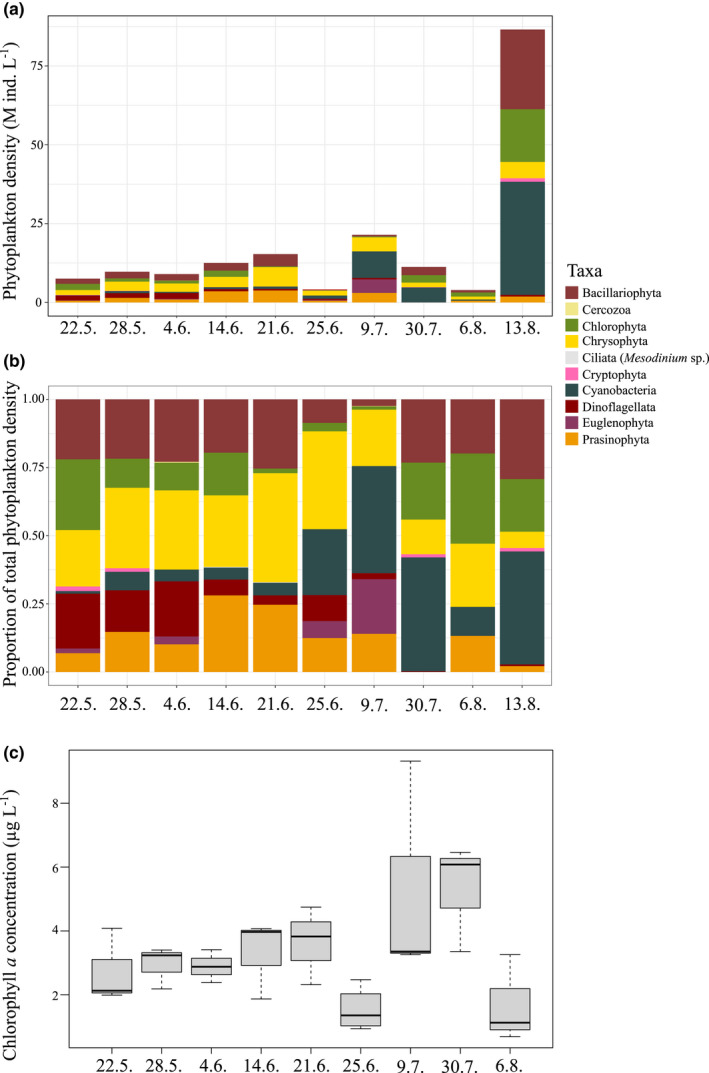
Phytoplankton taxa as (a) density (millions of individuals L^−1^) and (b) proportions of total phytoplankton density (in cell numbers) in May–August 2018. Data are semiquantitative as sample consists of cells >10 m. (c) Chl *a* concentration. The boxplots show the median (vertical line), interquartile range (IQR, the box), and minimum and maximum within 1.5 × IQR (“whiskers”)

**FIGURE 5:**
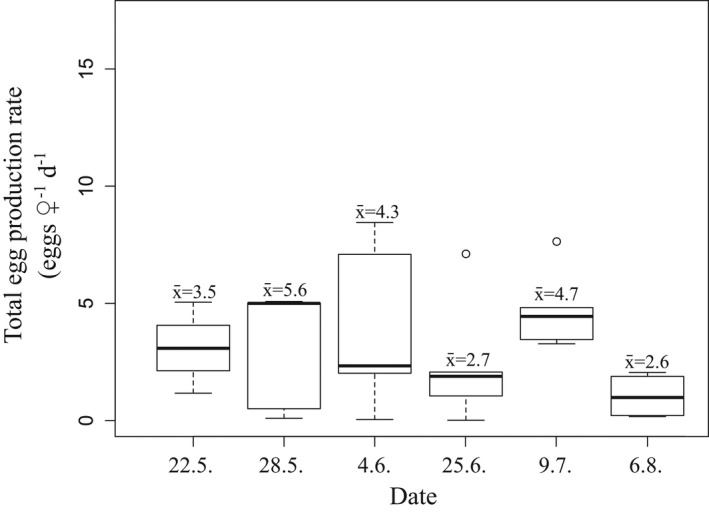
*In situ* egg production rate during May–August 2018. Mean values ($\bar{x}$) are shown above each boxplot. The boxplots show the median (vertical line), interquartile range (IQR, the box), and minimum and maximum within 1.5 × IQR (“whiskers”) and outliers (circle)

The May–June phytoplankton community (>10 m) was dominated by chrysophytes, which decreased in abundance after mid‐July (Figure 4a,b). Dinoflagellates were abundant from May to mid‐June and were few in August. Phytoplankton community was rich in diatoms throughout the sampling period (20–25% of the total phytoplankton between May 22 and June 21), except for July, when they formed 2% of the total phytoplankton (in cell numbers). Cyanobacteria abundance was low in the beginning of the season (1–7%) until the bloom started in late June. The peak in abundance was in mid‐August, when the proportion of cyanobacteria of the whole phytoplankton community reached 42%. Other taxa abundant throughout the summer were Chlorophyta and Prasinophyta. Overall food availability, measured as Chl *a* concentrations, peaked in mid‐July (5.3 g L^−1^), and the lowest concentration was detected 2 weeks earlier, on June 25 (1.5 g L^−1^, Figure 4c). Chl *a* concentration correlated positively with both temperature at 10 m (0.64, df = 28, *p* < .01) and proportion of cyanobacteria (0.68, df = 55, *p* < .01) (Figure 5).

### 3.3 Reproduction and female body size

**FIGURE 6:**
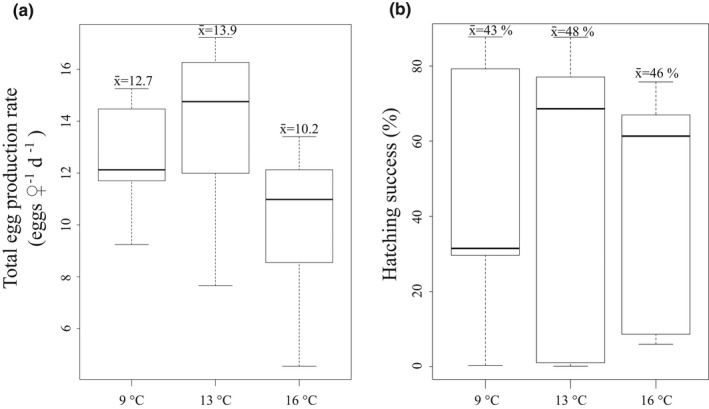
Egg production rate (a) and hatching success (b) in the experiment. Mean values ($\bar{x}$) are shown above each boxplot. The boxplots show the median (vertical line), interquartile range (IQR, the box), and minimum and maximum within 1.5 × IQR (“whiskers”) and outliers (circle)

The prosome length of adult females varied between 602 and 889 m during the experiment (Table 1). Mean P*_L_* did not differ between treatments or between sampling dates (Kruskal–Wallis rank sum test, *p* > .05). Also, offspring production rate did not correlate with P*_L_* of *Acartia* sp., indicating that neither egg production nor the biomarkers were affected by female body size. Egg production varied between 1.7 and 17.2 eggs female^−1^ d^−1^ in the experiment. As a comparison, the *in situ* egg production varied between 0.01 and 8.4 eggs female^−1^ d^−1^ (Figure 5). On average, egg production rate was nearly four times as high in the experiment than *in situ*. The egg hatching rate in the experiment varied between 0.1 and 87.7%, and the mean in control treatment was 43±13%, which is slightly less than in warmer temperature treatments: 48±14% in 13°C and 46±10% in 16°C (Figure 6). However, the difference between treatments was not significant. *In situ* egg production did not correlate with Chl *a* concentration (0.14, *p* > .05, df = 27) or temperature (−1.2, *p* > .05, df = 25).

**TABLE 1:** Body sizes (prosome length P*_L_*) of adult *Acartia* sp. copepods after egg incubation in the experiment, and egg production rates (EPR) with standard deviations

Treatment	Date	P*_L_* range (m)	*n*	Mean P*_L_* (m)	SD	Mean EPR	SD
9°C	14.6.	604–889	82	740	57	13.12	1.85
	16.6.	618–816	85	693	41	11.57	3.99
	21.6.	611–806	76	674	39	7.16	5.24
13°C	14.6.	608–853	73	709	54	6.37	4.19
	16.6.	629–769	83	688	31	16.40	1.13
	21.6.	602–787	66	681	38	8.55	1.22
16°C	14.6.	624–873	46	720	46	6.81	2.05
	16.6.	602–846	65	688	40	12.70	1.04
	21.6.	617–779	68	686	38	10.60	5.49

Copepod female survival during *in situ* egg incubations was usually high. Occasionally, one individual was found dead throughout the sampling season, except for 30 June when mortality was slightly higher (1–3 individuals out of 10). During experiments, around 1–4 out of 50 were dead after the experiments.

### 3.4 Oxidative stress in experimental setup

**FIGURE 7:**
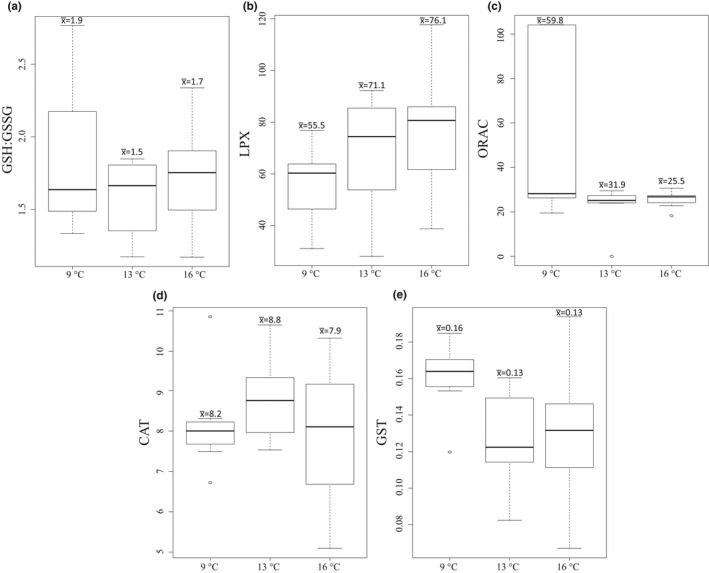
Biomarkers of oxidative stress in 9, 13, and 16°C treatments in the experiment: (a) GSH:GSSG ratio, (b) LPX (M cumene hydroperoxide equivalents mg^−1^ mg protein^−1^), (c) ORAC (M trolox equivalents mg^−1^), (d) CAT activity (mol min^−1^ mg^−1^), and (e) GST activity (mol min^−1^ mg^−1^). Mean values ($\bar{x}$) are shown above each boxplot. The boxplots show the median (vertical line), interquartile range (IQR, the box), and minimum and maximum within 1.5 × IQR (“whiskers”) and outliers (circle)

CAT activity ranged from 5.1 to 10.8 mol min^−1^ mg^−1^ during the experiment with an overall mean of 8.3 mol min^−1^ mg^−1^ (Figure 7). The range of ORAC readings observed during the experiment was 18–104 M trolox equivalents mg^−1^. The smallest variability of ORAC between replicates was observed in the 16°C treatment, which also had the lowest mean within treatments, whereas the highest measurements were in the control treatment (9°C). GST activity ranged from 0.06 to 0.2 mol min^−1^ mg^−1^ during the experiment, the overall mean being 0.14 mol min^−1^ mg^−1^. GST varied little between treatments. GSH:GSSG ratio was in general much lower in the experiment than in the field, varying from only 1.7 to 2.8. LPX showed the lowest measures in the control treatment and the highest in 16°C (mean 56–76 M cumene hydroperoxide equivalents mg^−1^ mg protein^−1^).

### 3.5 Effects of warming on reproduction and oxidative status

**FIGURE 8:**
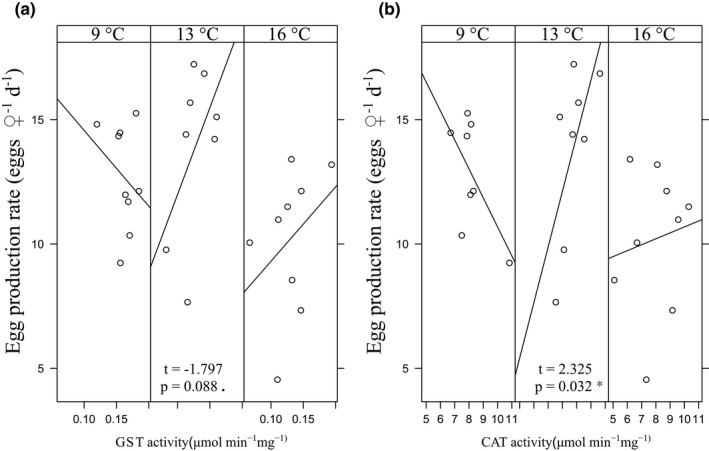
The interaction of temperature and (a) GST activity (b) CAT activity on offspring production rate in the experiment. The *p*‐values: *<.05, <.1

Warming had a negative effect on GST and ORAC in the experimental setup: GST activity differed between both treatments (13°C and 16°C) and the control (linear mixed models), whereas the change in ORAC was significant only in the 16°C treatment. Also, +7°C increase in temperature had a negative effect on offspring production rate (Table 2). The interaction of the CAT activity and the 13°C treatment had a significant effect on offspring production rate, whereas the treatment × GST activity interaction showed almost the same effect on both 13°C and 16°C treatments, shown as a statistical trend (Figure 8, *p* < .09).

**TABLE 2:** A list of response variables, fixed effects, and test results of chosen linear mixed models

Response variable	Fixed effects	Estimate ± SE	df	*t*‐value	*p*‐value
EPR	(Intercept)	11.8 ± 1.61	2	7.33	.02*
	(Intercept)	12.7 ± 1.41	2.93	9.01	.00**
	13°C	0.91 ± 1.08	21.01	0.85	.41
	16°C	−2.51 ± 1.04	20.99	−2.41	.03*
	(Intercept)	12.43 ± 2.04	7.46	6.09	.00***
	LPX	0.01 ± 0.02	16.06	0.45	.66
	13°C	0.43 ± 1.2	15.99	0.36	.73
	16°C	−3.18 ± 1.29	16	−2.45	.03*
	(Intercept)	20.8 ± 5.62	18.46	3.7	.00**
	CAT	−0.95 ± 0.67	17.22	−1.42	.17
	13°C	−20.16 ± 8.94	17.28	−2.25	.04*
	16°C	−10.58 ± 6.47	17.14	−1.64	.12
	CAT × 13°C	2.43 ± 1.04	17.31	2.33	.03*
	CAT × 16°C	0.95 ± 0.79	17.17	1.19	.25
	(Intercept)	13.71 ± 1.84	6.38	7.46	0***
	ORAC	−0.02 ± 0.02	20.2	−0.92	.37
	13°C	0.27 ± 1.28	20.11	0.21	.84
	16°C	−3.09 ± 1.22	20.04	−2.54	.02*
	(Intercept)	4.13 ± 8.82	20.62	0.47	.64
	GST	53.08 ± 53.33	19.5	1	.33
	13°C	16.06 ± 9.62	19.49	1.67	.11
	16°C	3.82 ± 9.64	19.59	0.4	.7
	GST × 13°C	−110.16 ± 61.29	19.49	−1.8	.09
	GST × 16°C	−35.82 ± 61.96	19.6	−0.58	.57
	(Intercept)	11.93 ± 3.38	19	3.53	0**
	GSH:GSSG	0.42 ± 1.73	17.79	0.24	.81
	13°C	−4.92 ± 5.84	17.14	−0.84	.41
	16°C	−4.32 ± 5.56	18.38	−0.78	.45
	GSH:GSSG × 13°C	3.81 ± 3.5	17.19	1.09	.29
	GSH:GSSG × 16°C	1.39 ± 3.1	18.49	0.45	.66
ORAC	(Intercept)	3.83 ± 0.18	22	20.78	>.01*
	13°C	−0.36 ± 0.28	22	−1.30	.2
	16°C	−0.60 ± 0.26	22	−2.31	.03*
GST	(Intercept)	0.16 ± 0.01	23	17.87	0***
	13°C	−0.03 ± 0.01	23	−2.62	.02*
	16°C	−0.03 ± 0.01	23	−2.5	.02*
*in situ* GST	(Intercept)	−3.19 ± 0.4	5.02	−7.87	.00***
	*in situ* temperature	0.10 ± 0.03	5.05	3.38	.02*

Note

Egg production rate (EPR) or biomarkers were used as response variables, while treatments (13 or 16°C), or *in situ* temperature from 10 m depth, were used as fixed effects. Interaction between fixed effects marked with ×. Sampling date was used as a random effect in all models. The *p*‐values: ***<.001, **<.01, *<.05, <.1.

### 3.6 Oxidative status in the field

**FIGURE 9:**
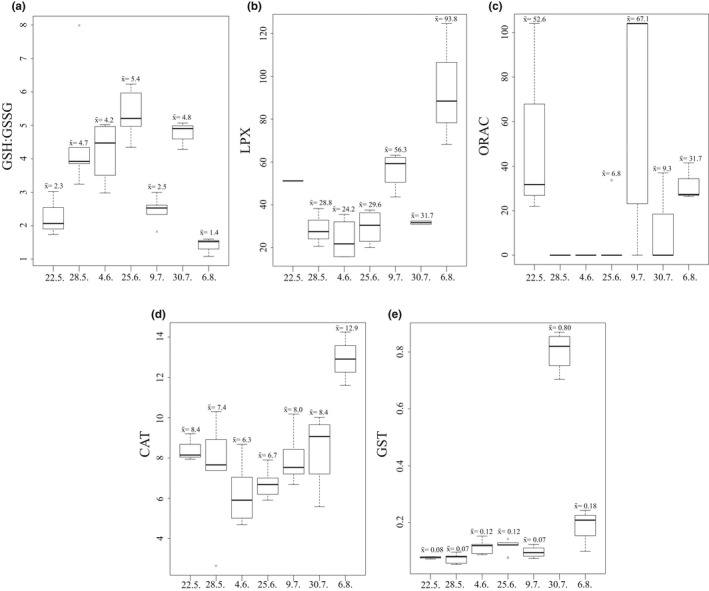
Biomarkers for oxidative stress and antioxidant defenses during summer 2018 in adult *Acartia* copepods. (a) GSH:GSSG ratio, (b) LPX (M cumene hydroperoxide equivalents mg^−1^ mg protein^−1^), (c) ORAC (M trolox equivalents mg^−1^), (d) CAT activity (mol min^−1^ mg^−1^), and (e) GST activity (mol min^−1^ mg^−1^). Mean values ($\bar{x}$) are shown above each boxplot. The boxplots show the median (vertical line), interquartile range (IQR, the box), and minimum and maximum within 1.5 × IQR (“whiskers”) and outliers (circle)

The activity of CAT varied between 2.6 and 10 mol min^−1^ mg^−1^, except for August 6, when the average activity peaked and the highest activities were recorded (14.3 mol min^−1^ mg^−1^, Figure 9). The average GST activity was the lowest in May and the highest on July 30, when GSH:GSSG ratio was also relatively high. In August, GST activity lowered considerably, while LPX was the highest and the GSH:GSSG ratio was the lowest. Overall, the GSH:GSSG ratio and LPX were negatively correlated during the field period (−0.7, *p* < .001). ORAC showed high variation between replicates; it varied between 0 and 104 M trolox equivalents mg^−1^, whereas the average was never higher than 67. We found no ORAC activity in weeks 22 and 23 (below detection level). In contrast to the experiment, temperature in 10 m had a positive effect on *in situ* GST (Table 2).

## 4 DISCUSSION

This study focused on the effects of warming on oxidative stress and reproduction of *Acartia* sp. in the Gulf of Finland. The work consisted of two parts: monitoring of *in situ* egg production and oxidative stress in May–August, and an experimental incubation, using three different temperature scenarios. The main finding in the experimental part was that temperature had negative effects on reproduction, GST and ORAC. In field monitoring, we saw that a strong heat wave in late July–August coincided with increasing oxidative stress.

### 4.1 Seasonality affecting reproduction in the field

The annual average seawater surface temperature (SST) at Storfjärden has increased by 1°C during 1927–2012 (Merkouriadi & Leppäranta, 2014). Previously, it has been reported that temperature influences the egg production rate (EPR) of *Acartia* sp. in the Baltic Sea (Diekmann et al., 2012; Koski & Kuosa, 1999; Peck & Holste, 2006; Vehmaa et al., 2012). In the current work, we did not detect direct temperature effects on reproduction in the field.

However, the *in situ* copepod EPR or eco‐physiological responses such as oxidative stress and AOX could still have been indirectly affected by food quality as chlorophyll *a* correlated significantly with the seasonal cyanobacteria abundance (Figure 4a). Toxic cyanobacteria are known to either cause oxidative stress in many organs of various species or alter the antioxidant system (Martins et al., 2017). Copepod *Acartia* spp. can feed on toxic cyanobacteria *Nodularia* (Engström‐Öst et al., 2015), and the toxin nodularin can cause increased antioxidant defenses (e.g., GST) in *Gammarus* (Turja et al., 2014).

The copepods were not provided food during the 24‐h incubations, and this may have increased the variability between *in situ* and experimental EPR. On the other hand, comparison of EPR between field and laboratory was not the main aim of this paper. Tester and Turner (1990) have demonstrated that it takes 24 h for *Acartia* copepods to make eggs. Koski and Kuosa (1999), on the other hand, used 48 h as the length of experimental acclimation. *In situ* EPR was low throughout the season, despite available dinoflagellates, which are a high‐quality food source for *Acartia* sp. reproduction (Vehmaa et al., 2011). In July and August, accelerated warming, cyanobacteria blooms, and decreasing dinoflagellate abundance may have caused larger variability in *in situ* egg production between sampling occasions.

### 4.2 Glutathione cycle responds to increasing stress in the field

How temperature will affect mechanisms in the cell is still not well known, especially concerning processes associated with redox chemistry during natural conditions (Reviewed by Birnie‐Gauvin et al., 2017). Changes in oxidative status in adult *Acartia* sp. females were detected from several biomarkers in field‐collected animals. GSH:GSSG ratio and LPX correlated negatively, which was expected as high LPX and low GSH:GSSG ratio, indicate oxidative stress (Lesser, 2006; Lushchak, 2011). Interestingly, our GSH:GSSG ratios in the field were low compared to previous studies on *Acartia* sp.: Glippa et al. (2018) reported ratios reaching 14, which is nearly three times higher than the highest mean ratio in this study (5.4 in June 25, see Figure 9). They also reported in general higher ORAC, more than ten times higher, considering that the animals were residing in approximately the same temperature conditions.

We found a positive response of increasing temperature on *in situ* GST, which is logic as the GST activity peaked in high temperatures (22–25°C from 0 to 20 m in July 30). GST is an enzyme that metabolizes organic hydroperoxides, which partially explains lower LPX during GST peak (Halliwell & Gutteridge, 2015). In fish, it has been shown that more acute exposure to warming caused higher antioxidant (AOX) levels, whereas when fish were exposed to more chronic type of temperature rise, AOX were close to baseline (Carney Almroth et al., 2015). Simultaneously with the peak in GST was a relatively high GSH:GSSG ratio in our data. Likely, this shows an effective response of glutathione cycle during temperature‐driven stress. This is in accordance with a previous suggestion that glutathione metabolism is efficient in copepods (Sokolova, 2013; Vuori et al., 2015).

The temperatures were higher than the average temperatures recorded in the past 85 years during the same month (Merkouriadi & Leppäranta, 2015). High temperature in the deeper parts of the water column is also highly relevant, since *Acartia* sp. are known to dwell close to the bottom during the day and near the surface at midnight in the study site (Almén et al., 2014). It has been observed that during moderate stress, AOX levels are overexpressed, and the redox balance can be maintained, thereby avoiding oxidative stress (Reviewed by Sokolova, 2013). It is possible that this phenomenon occurred in the beginning of the heatwave. Prolonged heatwave could have induced oxidative stress (Glippa et al., 2018; Kim et al., 2015; Vehmaa et al., 2013; Won et al., 2015), affecting *Acartia* sp. in the following week, when LPX values tripled, GSH:GSSG ratio declined from 4.8 to 1.4, and GST level lowered, too. Simultaneous to the highest LPX, CAT activity peaked. The peak of CAT suggests accumulated H_2_2O_2_ and thus oxidative stress; glutathione peroxidase typically activates in first line to remove smaller amounts of H_2_O_2_ (Costantini, 2014).

To conclude, proposed environmental stress caused by temperature increase, accompanying changes in oxygen, pH, and cyanobacteria, led to oxidative stress in *Acartia* copepods. Furthermore, our data show that the glutathione cycle of *Acartia* sp. (including GSH and GST) responds strongly to increasing stress. This is in accordance with a previous suggestion that glutathione metabolism is efficient in copepods (Vuori et al., 2015).

### 4.3 Negative temperature effect on reproductive rate, ORAC, and GST in the experiment

We found a significant negative effect of 16°C treatment on egg production rate. Vehmaa et al. (2013) found that a 3°C temperature increase (from 17 to 20°C) had a negative effect on egg viability and hatching rate, but not on egg production. Despite negative effects on reproduction, increased temperature has positively affected the abundance of *Acartia* sp., observed from a long‐term monitoring data in a southwest coast of Finland in 1967–2013 (Mäkinen et al., 2017).

Our work shows that a temperature increase of 4–7°C is tolerable to *Acartia* sp., proving its robustness and that it does not cease to reproduce in higher temperatures. The optimal temperature for *A*. *bifilosa* in light of reproduction is approximately 13–18°C (Koski & Kuosa, 1999), implying that our study was conducted within the tolerance range of *A*. *bifilosa*. A small decrease (−2.5 eggs female^−1^ d^−1^) in modeled reproduction rate in +7°C temperature treatment is important to note, but the fact that it is a relatively small decrease in EPR proves that *Acartia* may be a good survivor in the warmer future seas. The effect of temperature on oxidative stress was detected in GST, which was negatively affected by 13°C and 16°C treatments. Another biomarker for antioxidant defense, ORAC, had a negative response in 16°C treatment. This is partly in contrast with results by Vehmaa et al. (2013) whose experiment showed that temperature increase of 3°C had a positive effect on ORAC and oxidative damage in *Acartia* sp. It is possible that the generally high oxidative stress levels observed in all treatments (including the control) may have hindered the differences between treatments. Unknown factors in the experiment, in addition to elevated temperatures, may have induced stress, too. However, we may exclude shortage of food and oxygen depletion from these factors because copepods were well fed during the incubation, and a normoxic level of dissolved oxygen was recorded throughout the experiment. Normoxia is here >6 g L^−1^, according to Diaz (2016) in freshwater environments. The mortality during the experiment was relatively low (max. 8%), but there are several possible reasons for mortality, such as during the incubation ending process, where they were sieved and separated from the eggs, or pipetting.

Egg production rate was significantly affected by the interaction of the 13°C treatment and CAT activity, and also, the similar result for GST was almost significant. Interestingly, at 9°C both CAT and GST showed (nonsignificant) negative trends with egg production rate, while in 13°C (significant) and in 16°C (nonsignificant), the trends were positive. This suggests that temperature has affected the relationship between reproduction and antioxidants, and that there is a possible trade‐off between reproduction and oxidative stress in higher temperatures. A trade‐off between these two traits has been emphasized multiple times in the literature in copepods (Garzke et al., 2016; Rodríguez‐Graña et al., 2010; Vehmaa et al., 2013), and also widely in the animal kingdom (Metcalfe & Alonso‐Alvarez, 2010). However, it has to be kept in mind that animals have mechanisms in coping with stress: Repeated (but not continuous) stress may protect animals from further damage by ROS due to hormetic effects (Hood et al., 2018). Furthermore, female copepods have been suggested to be able to transfer part of the accumulated oxidative damage into offspring (Rodríguez‐Graña et al., 2010).

## 5 CONCLUSIONS

We showed that *Acartia* sp. females can tolerate an increase of 4–7°C to ambient temperature (9°C) and were able to reproduce in the experimental conditions with only a small decrease of egg production rate observed in the warmest treatment. Biomarkers of oxidative stress and antioxidant defense showed clear progression during the productive season in summer 2018 when oxidative stress increased in August, possibly due to seasonal effects, such as cyanobacteria blooms, and temperatures that increased above the optimum. Glutathione cycle had a clear response to increasing stress and possibly had an important role in preventing oxidative damage; lipid peroxidation and ratio of reduced and oxidized glutathione were negatively related throughout field season and in the experiment. The role of glutathione‐s‐transferase in antioxidant defense was shown as increased activity in the field when stress was introduced, and catalase activity peaked when the stress level was at its highest. In addition to temperature, food quality at the sampling site Storfjärden was probably an important factor affecting *in situ* egg production rate at least in May–June, when the water column was rich in dinoflagellates. Possibly, the combined effect of increased temperatures, high abundance of cyanobacteria, and low abundance of dinoflagellates caused higher variability in *in situ* egg production rate in July–August. Our data suggest a possible trade‐off between antioxidant defense and reproduction.

Finally, the interaction of temperature and salinity on reproduction still needs further studies. *Acartia* sp. are both eurythermal and euryhaline, but their tolerance to osmotic stress depends on temperature (Diekmann et al., 2012). The freshwater input to the Baltic Sea is increasing in future (HELCOM, 2021; Meier et al., 2012), and the typical salinity in our study area is often below the optimum salinity range 7–16 of *Acartia* sp. (Dutz & Christensen, 2018). Salinity is an important abiotic factor for brackish‐water copepods, and even small salinity changes may have surprising effects on oxidative status in copepods (Cailleaud et al., 2007; Martínez et al., 2020).

## CONFLICT OF INTEREST

The authors declare no conflicts of interest.

## AUTHOR CONTRIBUTIONS

**Ella Wilhelmina von Weissenberg:** Data curation (lead); formal analysis (lead); investigation (equal); methodology (equal); software (lead); validation (lead); visualization (lead); writing—original draft (lead); writing—review and editing (lead). **Anna Elina Jansson:** Conceptualization (equal); data curation (equal); formal analysis (supporting); funding acquisition (equal); investigation (equal); methodology (equal); project administration (equal); resources (supporting); software (supporting); supervision (equal); validation (supporting); visualization (supporting); writing—original draft (supporting); writing—review and editing (supporting). **Kristiina A. Vuori:** Data curation (equal); investigation (equal); methodology (equal); resources (supporting); visualization (supporting); writing—review and editing (supporting). **Jonna Engström‐Öst:** Conceptualization (equal); data curation (equal); formal analysis (supporting); funding acquisition (equal); investigation (equal); methodology (equal); project administration (lead); resources (lead); software (supporting); supervision (equal); validation (supporting); visualization (supporting); writing—original draft (supporting); writing—review and editing (supporting).

## Data Availability

Data used in this study were deposited in the Dryad Digital Repository: https://doi.org/10.5061/dryad.3bk3j9km3.
